# Polyvalent phage GSP004 recognizes O-antigen polysaccharide receptors in *Salmonella* and *Escherichia coli* through tail fiber protein ORF208

**DOI:** 10.1128/jvi.00810-25

**Published:** 2025-11-18

**Authors:** Dongyang Gao, Shenyu Pang, Yuanhang Zhao, Shunyuan Pan, Xiangyu Kong, Jun Song, Dongbo Sun

**Affiliations:** 1 College of Animal Science and Veterinary Medicine, Heilongjiang Bayi Agricultural University 91625, Daqing, China; 2 China Key Laboratory of Bovine Disease Control in Northeast China, Ministry of Agriculture and Rural Affairs, Daqing, China; 3 Heilongjiang Provincial Key Laboratory of Prevention and Control of Bovine Diseases, Daqing, China; Michigan State University, East Lansing, Michigan, USA

**Keywords:** polyvalent phage, *Salmonella*, *Escherichia coli*, receptor, receptor-binding proteins

## Abstract

The majority of phages are capable of lysing only specific bacterial strains within a single species, and polyvalent phages with cross-genus lytic capability are relatively rare. In this study, we isolated a polyvalent phage, GSP004, from swine farm wastewater, which exhibited cross-genus lytic activity against multiple *Salmonella* serovars and *Escherichia coli* (*E. coli*) O157:H7. Morphological classification assigns GSP004 to the family *Myoviridae* within the order *Caudovirales*, while phylogenetic analysis of its genome identifies it as a member of the genus *Kuttervirus* in the family *Ackermannviridae*. The periodate/proteinase K assays confirmed bacterial surface polysaccharides as the host receptor targeted by GSP004. Combined with lipopolysaccharide (LPS) competitive adsorption assays, gene knockout strain spot/adsorption/dynamic lysis assays, and phage nucleic acid release experiments, we demonstrated that the O-antigen of LPS was the sole receptor for GSP004 to infect *Salmonella* and *E. coli* O157:H7, respectively. Subsequent characterization via protein competition adsorption, antibody blocking, and fluorescent labeling experiments identified the tail protein ORF208 as the receptor-binding protein (RBP) recognizing LPS O-antigens. Further studies revealed that phage GSP004 employs its tail protein ORF208 to recognize and bind to the LPS O-antigen of *Salmonella* and *E. coli* through distinct molecular mechanisms, thereby mediating cross-genus infection. This finding provides critical molecular insights into the interaction between polyvalent phages and their bacterial hosts.

**IMPORTANCE:**

Elucidating the molecular mechanism of cross-genus host recognition in polyvalent phages will provide a critical theoretical foundation for the rational design of broad-host-range phages. However, research on the cross-genus host recognition mechanisms of polyvalent phages remains scarce. Here, we isolated a polyvalent phage GSP004, which serves as an exemplary model for investigating the interaction mechanisms between such polyvalent phages and their bacterial hosts. Our study elucidates the molecular basis underlying the capability of GSP004 to simultaneously infect *Salmonella* and *E. coli* O157:H7 across genera. This study provides crucial molecular evidence for understanding the evolutionary strategies by which phages expand their cross-genus host range and establishes a theoretical foundation for the rational design of broad-host-range phage therapeutics.

## INTRODUCTION

Antimicrobial resistance is a growing global health concern for both animal and public health, threatening to complicate the treatment of infections worldwide (1, 2). In 2021, it was estimated that 4.71 million deaths worldwide were due to antibiotic resistance, including 1.14 million deaths directly attributable to antibiotic resistance (3). The number of antibiotic-resistant infections is growing at an alarming rate, making it imperative to find alternative therapies. Phage therapy is a potential candidate treatment, and there have been increasing reports of successful phage therapies (4, 5). However, further understanding of the process by which phages infect bacteria will help optimize phage therapy.

Bacteriophages (phages) are viruses that specifically infect bacteria and are present in all environments where bacteria occur. The most common phages consist of double-stranded DNA genomes that are anchored to the head of an icosahedron attached to a contractile or non-contractile tail with fibers (6). The tail is a major structure in the early stages of the viral life cycle, including phage adsorption to the host first, followed by injection of its genome into the host cell. Usually, phage adsorption is divided into two binding steps, reversible binding and irreversible binding. For example, in T4-like phages (7), the baseplate-associated long-tailed fibers first reversibly bind to lipopolysaccharide (LPS) or OmpC, causing a conformational change in the baseplate, which results in the short-tailed fibers irreversibly binding to LPS. The signal generated by this process of irreversible binding is transmitted to the phage head, which then triggers the release of DNA. However, some phages can also use tail proteins to bind directly to a single receptor, *e.g*., *Siphovirus* 9NA and *Podovirus* P22 phages use their tail spike proteins to recognize the O-antigen receptor of LPS, which starts the infection process (8, 9). The detailed mechanisms vary in different phages, but tailed phages use receptor-binding proteins (RBPs) at the distal end of the tail for phage-host interactions, where tail spikes, tail fibers or spike proteins function as RBPs by attaching the phage to the host-encoded receptors (10). Phage receptors in Gram-negative bacteria typically involve outer membrane proteins (OMPs), LPS, capsular polysaccharides (CPS), pili, and flagella, and these unique surface structures ensure phage binding to the correct host (11). Phage host range depends largely on receptor availability and receptor structure, and analyzing how RBP interacts with its receptor is crucial to unraveling the mystery behind phage host specificity. Many mechanisms of how some monovalent phage RBPs interact with the host have been reported, such as P22, Mu, PNJ1809-36, and Bp7 (8, 12–14). However, there are few reports on the mechanisms of how polyvalent phages interact with the host.

In this study, a polyvalent phage, GSP004, capable of simultaneously infecting multiple *Salmonella* serovars and *Escherichia coli* (*E. coli*) O157:H7, was isolated. Genomic phylogenetic analysis revealed its classification within the *Kuttervirus* genus, demonstrating close evolutionary relationships with polyvalent phages BSP101, LPEK22, GG32, and SenALZ1. However, the mechanism of interaction between this type of phage and host bacteria of different genera remains unclear. Our investigation identified that GSP004 achieves dual-host adaptation through a singular tail protein ORF208. This protein specifically recognizes and binds to the LPS O-antigen of *Salmonella* and *E. coli* through distinct molecular mechanisms, thereby enabling cross-genus infection. This finding contributes to a deeper understanding of polyvalent phage infectivity for efficient phage applications.

## MATERIALS AND METHODS

### Bacterial strains

*Salmonella* and *E. coli* strains utilized in this study are detailed in Tables S5 and S6. Clinically isolated *E. coli* strains were initially isolated and screened using Eosin Methylene Blue agar (EMB; Oxoid, UK), while *Salmonella* spp. were initially isolated and screened using Xylose Lysine Desoxycholate agar (XLD; Oxoid, UK). The suspected target colonies obtained from the primary screening were subjected to PCR molecular identification using their specific primers (Table S7). *Salmonella* isolates confirmed by PCR were further subjected to serotype identification using commercial O and H antigen antisera (Tianrun Bio-Pharmaceutical, Ningbo, China) according to the White-Kauffmann-Le Minor scheme (15). For subsequent experiments, bacterial cultures were propagated in Luria-Bertani (LB) broth at 37°C with continuous shaking (200 rpm). In addition, the knockout and complementary strains used in this study were constructed from our previous study (Table S4).

### Isolation and purification of phages

*S*. Enteritidis SE006 (GenBank accession no. CP099973.1) (16) was used as the host for phage isolation. Wastewater samples were collected at a pig farm in Daqing, China. Phage isolation was performed using a double-layer plate method with slight modification as described previously (17). Briefly, the wastewater samples were centrifuged at 8,000 × *g* for 5 min at room temperature, and the supernatant was filtered using a 0.22-µm filter to ensure sterility. The filtrate was mixed with the *S*. Enteritidis SE006 and LB broth, then incubated at 37°C for 12 h to enrich the phage. After enrichment, the mixture was centrifuged and filtered again to obtain the phage-enriched filtrate. The gradient-diluted filtrate was added with the host bacteria to 5 mL LB soft agar (0.7% [wt/vol] agar) and poured onto LB agar plates (1.5% [wt/vol] agar). After 12 h of incubation at 37°C, single plaques were picked and resuspended in SM buffer (100 mM NaCl, 10 mM MgSO_4_, 50 mM Tris-HCl, pH 7.5). The resuspension was mixed with fresh host bacteria and plated again, and three rounds of purification were performed to obtain a single phage. The final purified phage was stored at 4°C or −80°C (containing 25% glycerol protectant).

### Determination of host range and efficiency of plating

The host range of phage GSP004 was determined by the efficiency of plating (EOP) as described previously (18). In brief, freshly propagated phage GSP004 (10^9^ PFU/mL) was serially diluted 10-fold (10^−3^ to 10^−9^) in SM buffer. Aliquots (10 µL) of each dilution were added dropwise to bacterial lawns of test strains. Following overnight incubation at 37°C, EOP was calculated based on the number of plaques formed (EOP, phage titer of test bacteria/phage titer of host bacteria).

### Transmission electron microscopy

Purified phage lysate (10⁹ PFU/mL) was prepared for TEM analysis. Subsequently, a drop of phage lysate was dropped onto a copper grid containing carbon (Carbon Type-B 200 mesh; Beijing Zhongjingkeyi Technology Co., Ltd., Beijing, China) and staining was performed with 2% (wt/vol) phosphotungstic acid (pH 6.5) as described previously (19). Images of phages were captured using transmission electron microscopy (H-7650, Hitachi, Tokyo, Japan) with an acceleration voltage of 100 kV.

### Genome analysis and phylogenetic analysis

The concentrated phage suspensions were treated with DNase I and RNase A to remove residual host-derived nucleic acids. Phage DNA was subsequently extracted using the Viral Genome DNA Extraction Kit (Omega Bio-Tek Inc., Doraville, GA, USA). Whole-genome sequencing of phage GSP004 was performed on the Illumina MiSeq platform (San Diego, CA, USA), with raw reads assembled using SPAdes v3.15.2 (20). Genome annotation was conducted through the RAST server (http://rast.nmpdr.org/) and cross-verified via BLASTp analysis (https://blast.ncbi.nlm.nih.gov/Blast.cgi). Circular genome visualization was generated using the Proksee platform (https://proksee.ca/). To determine the classification of phages, sequences belonging to different phage terminase large subunits were downloaded from the NCBI database based on the classification report of the International Committee on Taxonomy of Viruses (ICTV). A phylogenetic analysis of the terminase large subunit of phage GSP004 was then performed using MEGA 11 with the neighbor-joining method (21). Intergenomic similarity levels were calculated via VIRIDIC (http://rhea.icbm.uni-oldenburg.de/VIRIDIC) (22) and graphically optimized by Chiplot (https://www.chiplot.online/index.html). Genome comparison of phage GSP004 was performed using EasyFig software (23). The presence of potential virulence and antibiotic resistance genes in the phage genome was detected by CARD (https://card.mcmaster.ca/analyze/rgi) (24). Manually verify the presence of lysogen-associated proteins in the phage genome using the already annotated phage genome.

### Identification of the receptor type of phage GSP004

The type of receptor targeted by the phage was determined by periodate (IO_4_^−^, destroying LPS) and proteinase K (destroying OMPs) treatment tests (25). After the bacteria were cultured to the logarithmic growth stage, 1 mL of the bacterial suspension was washed with PBS to remove metabolic byproducts. The bacterial pellet collected after centrifugation was resuspended in 1.5 mL of sodium acetate (50 mM; pH 5.2) or sodium acetate containing either 10 mM or 100 mM IO_4_^−^, followed by dark incubation at 37°C for 2 h. Alternatively, the bacterial pellet collected after centrifugation was resuspended in 10 or 20 mg/mL proteinase K solution, with control groups lacking proteinase K, and incubated at 37°C for 3 h. After treatment, the bacteria were washed again with PBS before determining the phage adsorption rates.

### Inhibitory effect of LPS on adsorption

LPS was extracted from *S*. Enteritidis SE006 or *E. coli* ATCC 35150 using the LPS Extraction Kit (Bestbio, Shanghai, China) for subsequent experiments. Bacterial strains were cultured to logarithmic phase, and the metabolites were washed with PBS. A 200-µL aliquot of a phage suspension (10^9^ PFU/mL) was mixed with LPS solution at different concentrations (final concentrations: 10, 1, and 0.1 µg/mL), while the control group received buffer without LPS. The mixtures were incubated at 37°C for 15 min. Subsequently, 200 µL of a bacterial culture (10⁸ CFU/mL) was added to each mixture, followed by a further incubation at 37°C for 15 min. Finally, the mixtures were centrifuged at 12,000 × *g* for 1 min at 4°C. The supernatant was used to measure the plaque number and the adsorption percentages were calculated [1 − (phage titer of supernatant after cells were removed/phage titer of control reaction mixture without bacterial cells)] ×100%.

### Phage spotting assay

The receptors of host bacteria recognized by phage were further analyzed using constructed SE006 and ATCC 35150 gene knockout strains (Table S4). Ten-fold serial dilutions of phage lysates were prepared in SM buffer. For each strain, 10-µL aliquots of each dilution were spotted onto lawns of wild-type or knockout strains overlaid on double-layer LB agar plates. Plates were incubated at 37°C for 12–16 h to allow plaque development.

### Phage adsorption assay

Wild-type and knockout strains were cultured in LB medium at 37°C until the density reached 10^8^ CFU/mL. Then, phage GSP004 was added according to the optimal MOI. The mixture was centrifuged at 12,000 × *g* for 1 min after the addition of phage for 10 min. The supernatant was collected, and phage titer was quantified by the double agar overlay method. Adsorption percentages were calculated.

### Dynamic lysis activity of phage against host bacteria

Wild-type, knockout strain, and complementary strains were cultured to the logarithmic growth stage. A mixture of 100 µL bacterial suspension and 100 µL phage suspension (at the optimal MOI) was added to a 96-well plate. For controls, 100 µL bacterial suspension was mixed with 100 µL LB medium (positive control), and sterile LB medium served as the negative control. The 96-well plates were incubated at 37°C by shaking in the Feyond-A300 Multi-function enzyme immunoassay analyzer (Allsheng, Hangzhou, China), and the OD_600_ value was monitored every 30 min for 12 h.

### Fluorescence DNA ejection assay

The *in vitro* DNA ejection was detected using the Yo-Pro fluorescence assay, as previously described (8, 26, 27). Phage GSP004 (10^9^ PFU/mL) was mixed with LPS (final concentration: 10 µg/mL) derived from either *S*. Enteritidis SE006 or *E. coli* ATCC 35150, followed by incubation at 37°C. Throughout the incubation period, the mixture was maintained in the presence of 1 µM Yo-Pro-1 Iodide, and the fluorescence intensity was measured at 60-s intervals for a total duration of 6,000 s. At the end of each experiment, DNase I (10 µg/mL) was added as a control for DNA accessibility.

### Protein expression and purification

The genes encoding ORF206, ORF207, ORF208, and ORF209 were amplified by specific primers ORF206-F/R, ORF207-F/R, ORF208-F/R, and ORF209-F/R, respectively (Table S3). The resulting PCR products and the pET28a vector were digested with *Eco*RI and *Xho*I restriction enzymes. The digested fragments were then ligated into the linearized pET28a vector at the corresponding *Eco*RI/*Xho*I sites, generating the recombinant plasmids pET-28a-ORF206, pET-28a-ORF207, pET-28a-ORF208, and pET-28a-ORF209. To construct the EGFP-tagged recombinant plasmid pET-28a-ORF208-EGFP, the EGFP gene was amplified with primers ORF208-EGFP-F/R (Table S3). Both the EGFP amplicon and the pET-28a-ORF208 plasmid were digested with *Eco*RI and *Bam*HI, followed by ligation at the corresponding sites. All recombinant plasmids were verified by DNA sequencing.

The confirmed plasmids were transformed into *E. coli* BL21 (DE3) competent cells. Recombinant protein expression was induced by adding 0.5 mM IPTG to the cultures followed by incubation at 16°C for over 16 h. Cells were harvested by centrifugation at 10,000 × *g* for 10 min at 4°C, resuspended in PBS, and lysed via ultrasonication. The lysate was then purified by Ni-NTA Beads 6FF gravity columns. Finally, the BCA protein quantitative kit (Beyotime Biotechnology, Shanghai, China) was used to determine its concentration.

### Protein competition assay

To test the ability of recombinant proteins to compete with phages for adsorption to host bacteria, we performed assays as previously described (14). First, 200-µL purified recombinant protein (2 mg/mL) was mixed with 200-µL bacteria (10^8^ CFU/mL) and incubated at 37°C for 15 min. Subsequently, 100-µL phage (10^9^ PFU/mL) was added to the mixture after incubation at 37°C for 15 min. Afterward, the mixture was centrifuged at 12,000 × *g* for 1 min. The number of phage spot formations in the supernatant was then determined, and adsorption percentages were calculated.

### Polyclonal antibody blocking assay

Polyclonal antibody against ORF208 was prepared by immunizing New Zealand White rabbits with the purified ORF208 protein, following the previously described method (14). To determine the blocking effect of antibodies on phage infectivity, a neutralization assay was performed. Anti-ORF208 antibody was co-incubated with phage GSP004 (10^9^ PFU/mL) at 37°C for 20 min. Following the incubation, the residual infectivity of the phage was evaluated by determining its titer using the double-agar-layer plaque assay. All animal procedures were performed following the guidelines approved by the Animal Experiment Ethical Committee of Heilongjiang Bayi Agricultural University (approval no. DWKJXY2024038).

### Detection of fluorescent ORF208 binding to LPS

The detection method of ORF208 combined with LPS was slightly modified according to previous methods (12). To test whether the RBP of phage GSP004 could bind to the LPS extracted from *S*. Enteritidis SE006 or *E. coli* ATCC 35150, recombinant pET-28a-ORF208-EGFP vectors were constructed and proteins were then expressed and purified. For the assay, 200-µL purified ORF208-EGFP protein (2 mg/mL) was mixed with 200-µL wild-type strains (SE006 or ATCC 35150), knockout strains, or complementary strains (10^8^ CFU/mL) and incubated at 37°C for 15 min. After incubation, the bacteria were collected by centrifugation at 3,000 × *g* for 10 min, washed with PBS three times, and observed by fluorescence microscope.

### Bioinformatics analysis of ORF208

The domain architecture of ORF208 was analyzed using the Basic Local Alignment Search Tool (BLAST), Inter-ProScan software (http://www.ebi.ac.uk/Tools/InterProScan), and the protein families (Pfam) database (http://pfam.xfam.org/search). The amino acid sequence alignments of ORF208 were performed using Clustal Omega and GeneDoc. The three-dimensional (3D) structure of ORF208 was predicted through the AlphaFold Protein Structure Database (https://alphafold.com).

### Degradation of LPS with ORF208 for SDS-PAGE analysis

To test whether ORF208 can degrade LPS O-antigen, an SDS-PAGE analysis was performed. Purified ORF208 protein (2 mg/mL) was mixed with an equal volume of LPS (40 µg/mL) extracted from either the SE006 or ATCC 35150 strains, and the mixture was incubated at 37°C for 12 h. The reaction was stopped by the addition of proteinase K, which was followed by incubation at 95°C for 10 min. LPS was loaded onto SDS-PAGE. Digestion products were shown by 15% SDS-PAGE silver staining (Thermo Fisher Scientific, Cleveland, OH, USA).

### Statistical analysis

All data are presented as the mean ± standard deviation (SD) of at least three independent experiments. Data were analyzed by one-way analysis of variance (ANOVA) followed by Dunnett’s post hoc test for comparisons with a control group. All analyses were performed using OriginPro (OriginLab, Northampton, MA, USA). A *P* value below 0.05 was considered statistically significant, and significance levels in the figures are denoted by asterisks (*P < 0.05, **P < 0.01, ***P < 0.001; ns, not significant).

## RESULTS

### Host range of phage GSP004

Phage GSP004 was initially isolated from wastewater samples using *S*. Enteritidis SE006 as the host strain. Analysis of the host range of GSP004 revealed broad-host-range lytic activity, targeting not only 47 out of 59 tested *Salmonella* strains (spanning multiple serotypes) but also 21 *E. coli* O157:H7 strains (Table S1). This intergeneric lytic activity demonstrates that GSP004 is a polyvalent phage with cross-genus infectivity.

### Morphological observation of phage GSP004

Plaque morphology observations revealed that GSP004 formed clear plaques with a diameter of approximately 1 mm (Fig. S1A). Electron microscopic examination (TEM) demonstrated that GSP004 possessed an icosahedral head (65 ± 1.6 nm) and a long contractile tail (166 ± 0.9 nm) as shown in Fig. S1B. Based on morphological characteristics observed by TEM, phage GSP004 was classified into the order *Caudovirales*, family *Myoviridae*.

### General genomic features

**Fig 1:**
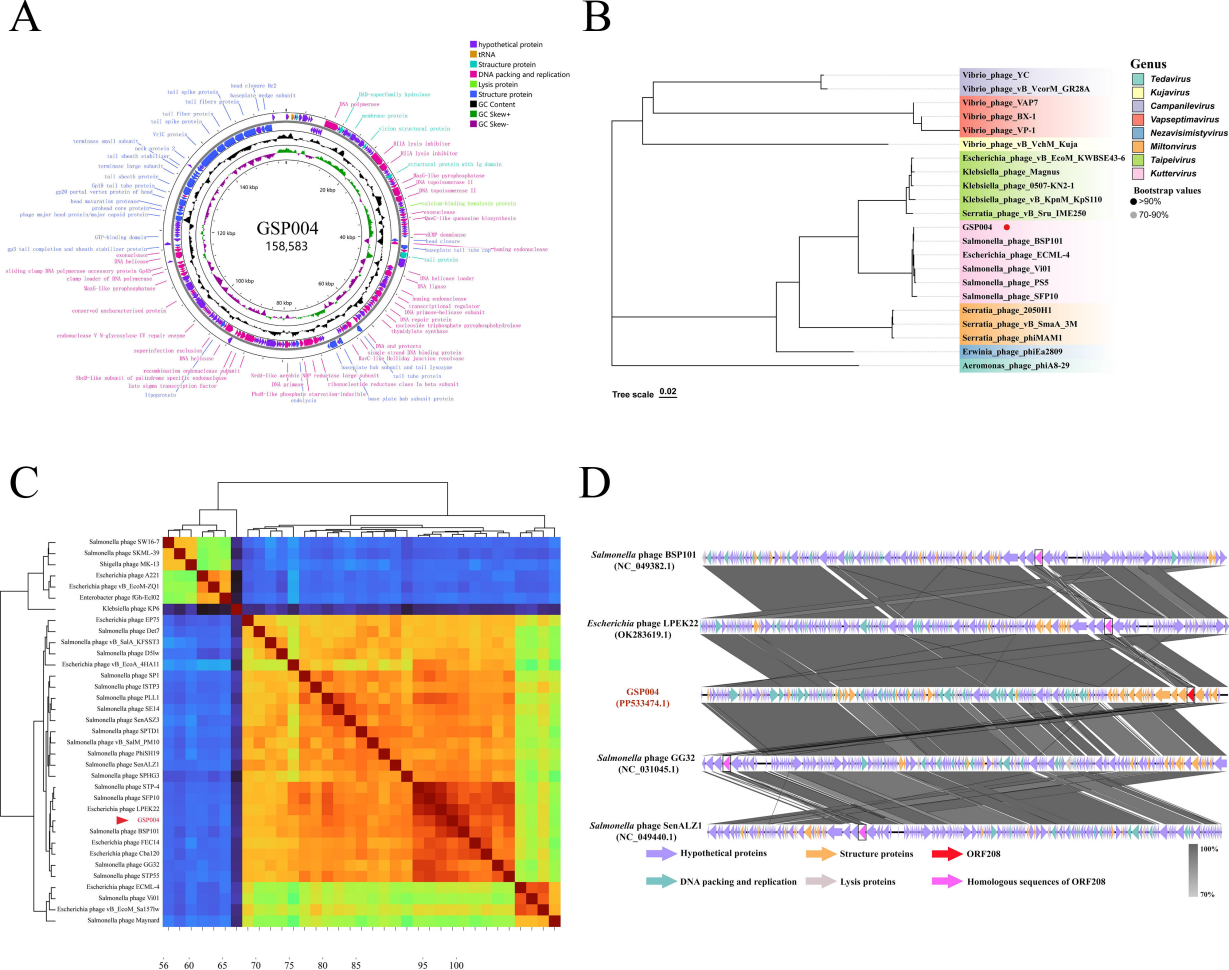
Genomic analysis of phage GSP004. (**A**) Circular genome map of phage GSP004. The functions of the open reading frame (ORF) are represented with specific colors according to their predicted functional categories. (**B**) Phylogenetic tree of phage GSP004 based on the sequence of the terminase large subunit. (**C**) Comparative genomics of phage GSP004 with other related phages (*n* = 34). Whole genome alignment is visualized as a heat map for average nucleotide identity. The closest relatives are indicated by phylogenetic trees. (**D**) Comparative linear genome map of phage GSP004 with polyvalent phages BSP101 (98.37%), LPEK22 (98.90%), GG32 (97.60%), and SenALZ1 (97.28%). The genome annotations are color-coded according to function, as shown in the key.

The phage genome comprised a circular double-stranded DNA of 158,583 bp with a G + C content of 44.57% (GenBank accession no. PP533474.1) (Fig. 1A). The genome encoded 4 tRNA genes and 214 putative open reading frames (ORFs) (Table S8).

The phylogenetic analysis based on the terminase large subunit indicated that GSP004 belonged to the *Kuttervirus* genus (Fig. 1B). Consistent with phylogenetic analysis, nucleotide-level homology analysis revealed close relationships with other *Kuttervirus* phages (Fig. 1C), with particularly high average nucleotide identities observed for BSP101 (98.37%), LPEK22 (98.90%), GG32 (97.60%), and SenALZ1 (97.28%) (Fig. 1D), all of which were able to simultaneously infect *Salmonella* and *E. coli* O157:H7. In addition, the genome analysis did not reveal any antibiotic resistance, virulence, or lysogeny-related genes, indicating that phage GSP004 could be a potential candidate for therapeutic applications.

### Phage receptor assay

**Fig 2:**
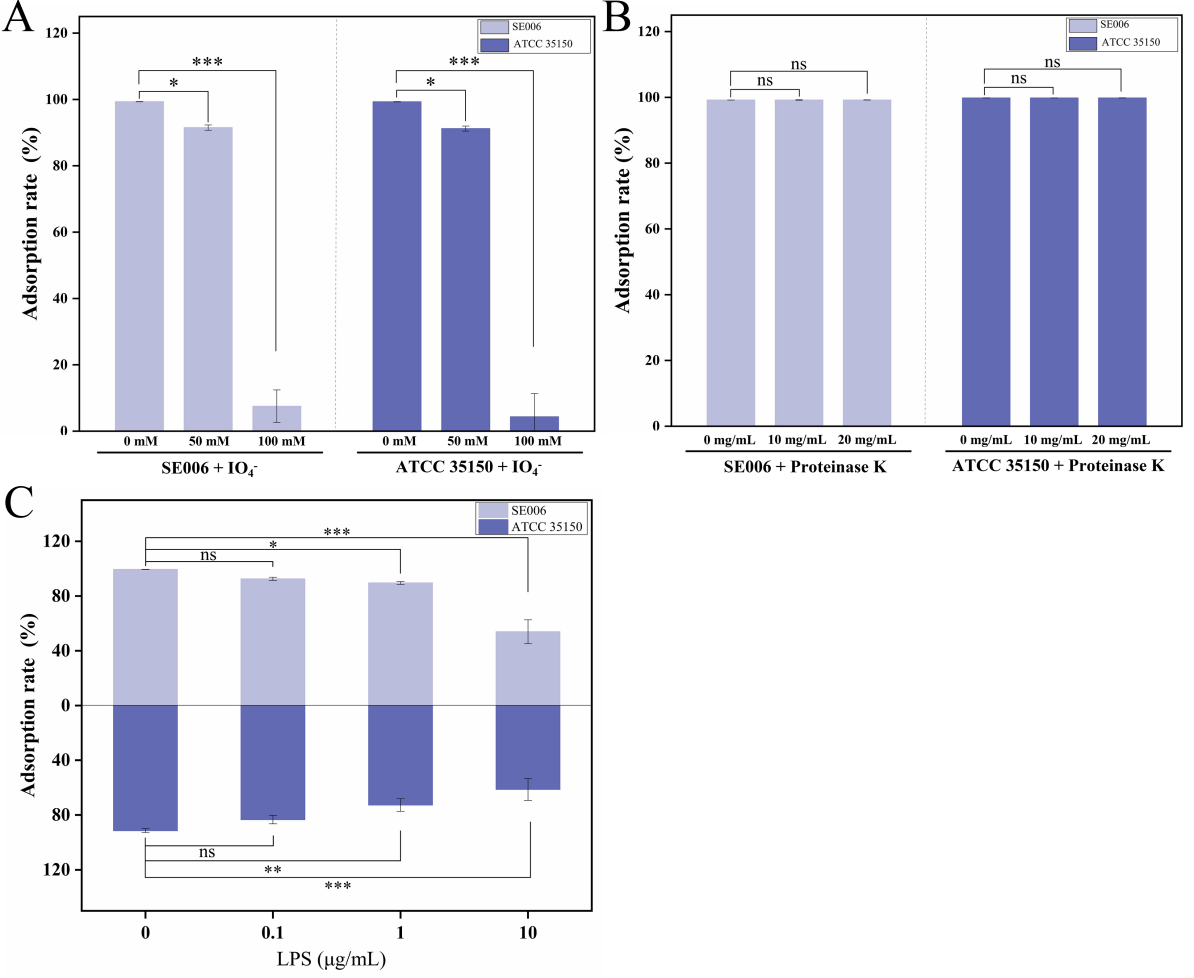
Identification of phage-targeted receptor types. (**A**) Adsorption of phage GSP004 to *S*. Enteritidis SE006 and *E. coli* ATCC 35150 treated with periodate (IO_4_^-^). (**B**) Adsorption of phage GSP004 to *S*. Enteritidis SE006 and *E. coli* ATCC 35150 treated with proteinase K. (**C**) Competitive inhibition of phage adsorption by LPS. Phage GSP004 was mixed with LPS (0–10 µg/mL) extracted from *S*. Enteritidis SE006 or *E. coli* ATCC 35150, and then its adsorption capacity to the host was assessed. *P < 0.05, **P < 0.01, ***P < 0.001; ns, not significant.

The bacterial surface primarily consists of OMPs and LPS, which are commonly utilized by phages as receptors. In order to identify the receptor for GSP004 recognition of host bacteria, *S*. Enteritidis SE006 and *E*. coli ATCC 35150 were treated with protease K (destroying OMPs) or periodate (destroying LPS), followed by phage adsorption assays. The results showed that there was a marked reduction in adsorption rate for both bacterial strains following periodate treatment (Fig. 2A and B), indicating that LPS is essential for phage attachment. Furthermore, in competitive inhibition assays, LPS extracted from these two bacterial strains was able to effectively block the adsorption of phage GSP004 to its host (Fig. 2C). Collectively, these findings indicate that LPS serves as the critical receptor enabling GSP004 to infect both *Salmonella* and *E. coli* O157:H7.

**Fig 3:**
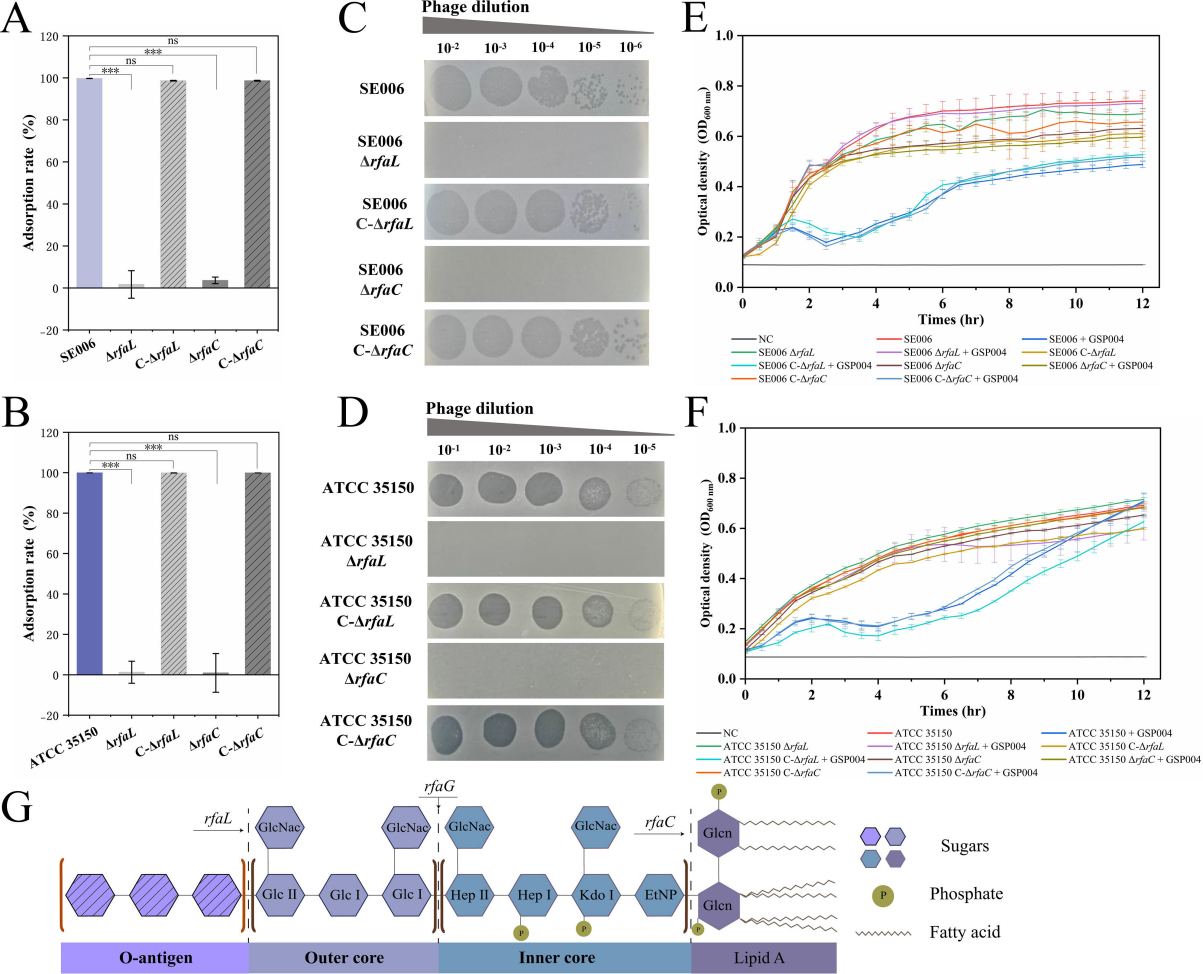
LPS O-antigen is the target receptor for phage GSP004. (**A, B**) Adsorption capacity of phage GSP004 to Δ*rfaL* (LPS O-antigen-deficient) and Δ*rfaC* (LPS core-deficient) mutants of *S*. Enteritidis SE006 (**A**) or *E. coli* ATCC 35150 (**B**). (**C, D**) Lysis efficiency of phage GSP004 against Δ*rfaL* and Δ*rfaC* mutants of *S*. Enteritidis SE006 (**C**) or *E. coli* ATCC 35150 (**D**) on solid agar medium. (**E, F**) Lysis kinetics of phage GSP004 against *S*. Enteritidis SE006 (**E**) and *E. coli* ATCC 35150 (**F**) mutants (Δ*rfaL*, Δ*rfaC*) in liquid culture. (**G**) Schematic diagram showing the structure of LPS and the various truncated mutants, modified from references (28, 29). ***P < 0.001; ns, not significant.

To further validate LPS as a receptor for phage GSP004, we performed adsorption assays and plaque formation tests using SE006 and ATCC 35150 strains harboring deletions in the LPS biosynthesis genes *rfaL* and *rfaC*. Phage adsorption assays demonstrated complete abolition of GSP004 binding to Δ*rfaL* and Δ*rfaC* mutants (Fig. 3A and B). Consistent with this, no plaques were observed following infection of bacterial lawns with these mutants (Fig. 3C and D). Moreover, *in vitro* growth kinetics revealed that phage GSP004 had no discernible impact on the proliferation of Δ*rfaL* or Δ*rfaC* cultures (Fig. 3E and F). These results suggest that LPS is indispensable for both phage adsorption and subsequent lytic activity.

The canonical LPS molecule consists of lipid A, core oligosaccharide, and O-antigen (Fig. 3G). Deletions in LPS biosynthesis genes produce truncated variants with distinct structural deficiencies. Specifically, Δ*rfaL* mutants retain an intact core oligosaccharide but lack the O-antigen, whereas Δ*rfaC* mutants exhibit profound truncation, presenting only lipid A with complete absence of both core oligosaccharide and O-antigen. The phage adsorption and lysis experiments described above show that truncation of the O-antigen alone affects phage adsorption and lysis of host bacteria, suggesting that the O-antigen is the major recognition region of phage GSP004.

### Phage GSP004 releases its DNA upon LPS incubation *in vitro*

**Fig 4:**
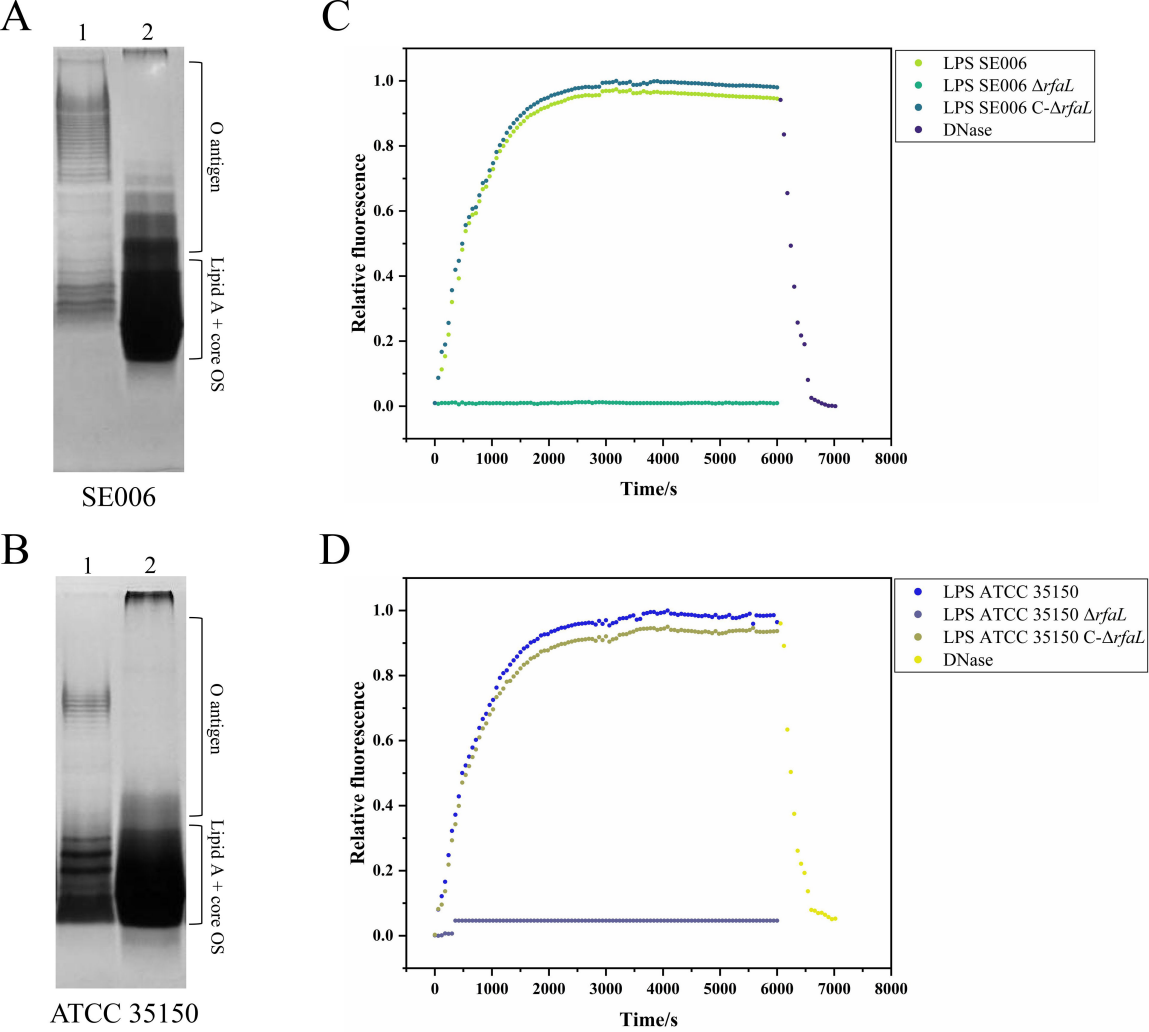
*In vitro* DNA ejection from phage GSP004 particles. (**A, B**) Silver staining analysis of LPS. Lane 1, LPS extracted from wild-type strains SE006 (**A**) or ATCC 35150 (**B**). Lane 2, LPS from Δ*rfaL* mutants of SE006 (**A**) or ATCC 35150 (**B**). The positions of the fully synthesized LPS, containing the attached O-antigen and the lipid A-core oligosaccharide (OS) precursor, are indicated. (**C, D**) *In vitro* monitoring of LPS-induced DNA ejection. Phage GSP004 was incubated with LPS from *S*. Enteritidis SE006 (**C**) or *E. coli* ATCC 35150 (**D**), and DNA release was quantified using a fluorescent DNA-binding dye. Increased fluorescence intensity after LPS treatment and decreased after the addition of DNase I, confirming that DNA was released from phage particles. The standard deviations (SDs) from three independent experiments were less than 1% of total fluorescence for every experiment.

To investigate whether LPS O-antigen as a binding receptor can trigger the release of phage DNA, we employed a fluorescent assay to monitor phage DNA ejection *in vitro*. The results demonstrated that incubation of phage GSP004 with LPS extracted from wild-type SE006 or ATCC 35150 strains elicited a time-dependent increase in dye fluorescence, plateauing after approximately 2,000 s at 37°C (Fig. 4). By contrast, no fluorescence enhancement was detected when phage GSP004 was incubated with LPS from O-antigen-deficient mutants (Δ*rfaL*). To validate that the fluorescence signal originated from phage DNA, DNase I was introduced post-signal saturation. This resulted in a rapid reversal of the fluorescence increase, confirming that the signal was attributable to phage-injected DNA. Collectively, these findings substantiate the LPS O-antigen as a specific binding receptor for phage GSP004. The above results further demonstrate that LPS O-antigen is a specific binding receptor for phage GSP004.

### Identification of receptor binding proteins of phage GSP004

**Fig 5:**
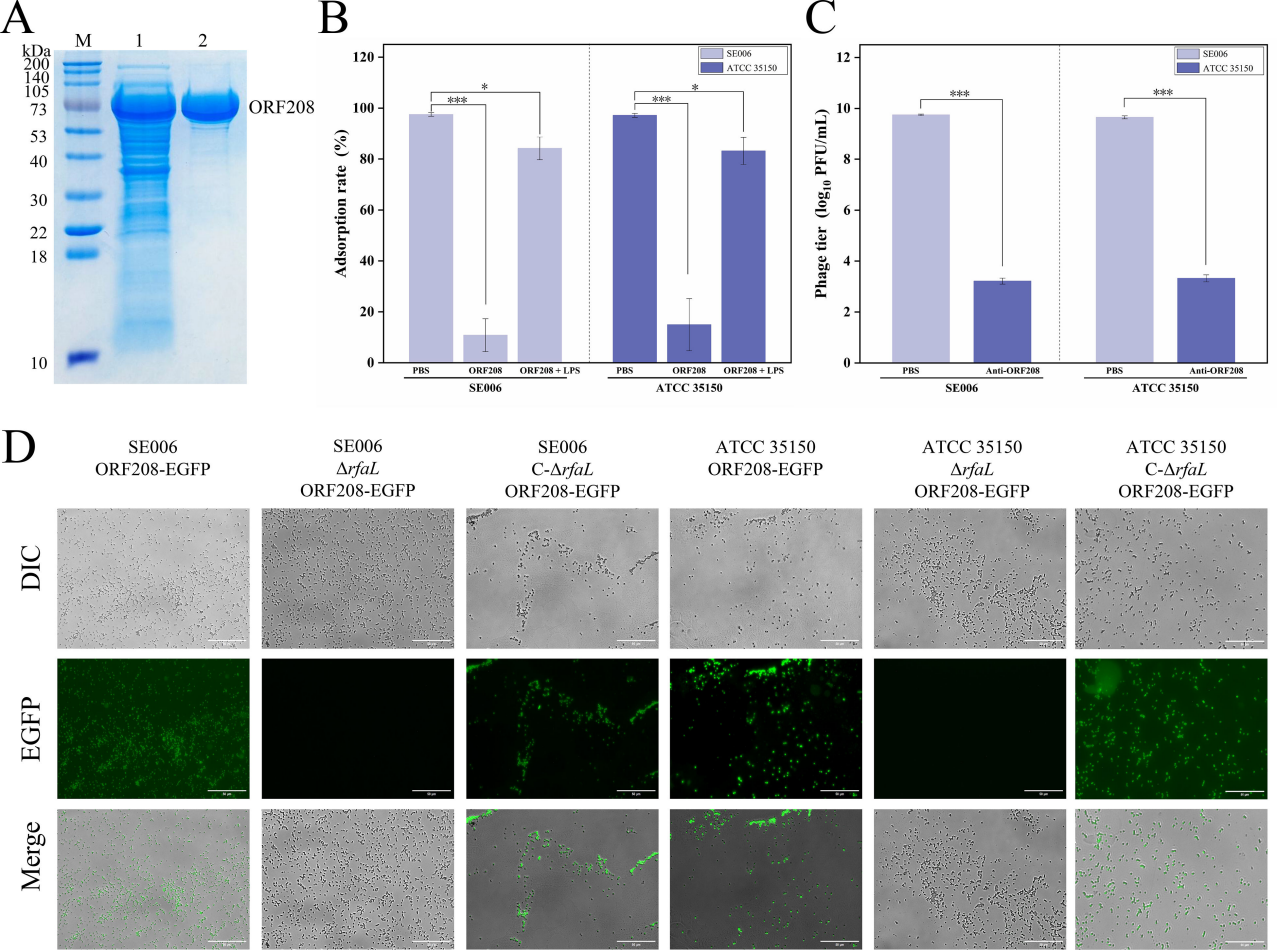
Prediction and identification of the RBP from phage GSP004**.** (**A**) Purification of recombinant ORF208 protein analyzed by SDS-PAGE with Coomassie blue staining. Lane M, Precision Plus Protein Dual Color Protein Marker (Bio-Rad). Lane 1, unpurified recombinant ORF208 protein. Lane 2, Ni-NTA affinity chromatography purified recombinant ORF208 protein. (**B**) Competitive binding assay of ORF208 protein to host bacteria. Pre-incubation of *S.* Enteritidis SE006 or *E. coli* ATCC 35150 with ORF208 reduced phage adsorption. Conversely, the addition of LPS restored adsorption by competing with ORF208 binding. (**C**) Antibody blocking assay. Pre-incubation of phage GSP004 with anti-ORF208 antibody significantly inhibited bacterial infectivity. (**D**) Fluorescence microscopy analysis of EGFP-tagged ORF208 binding to wild-type, Δ*rfaL* mutants, and Δ*rfaL*-complemented strains. EGFP-tagged ORF208 binds to wild-type and complemented strains, but not to Δ*rfaL* mutant. *P < 0.05, ***P < 0.001.

To identify phage-encoded receptor-binding proteins (RBPs), we first conducted bioinformatics analyses on four tail-associated proteins (ORF206, ORF207, ORF208, and ORF209) encoded by phage GSP004 (Fig. S2). These proteins were cloned, expressed, and purified using a prokaryotic expression system (Fig. 5A; Fig. S2). Adsorption inhibition assays revealed that incubation of recombinant protein ORF208 with phage GSP004 significantly reduced phage adsorption to SE006 or ATCC 35150, whereas addition of LPS restored adsorption efficiency (Fig. 5B). In contrast, purified recombinant proteins ORF206, ORF207, or ORF209 had no detectable effect on phage adsorption. Furthermore, pretreatment of phage GSP004 with a polyclonal antibody against ORF208 resulted in a marked reduction in plaque formation upon infection of strains SE006 or ATCC 35150 (Fig. 5C). These results suggest that the tail protein ORF208 acts as an RBP during phage adsorption.

To validate ORF208-O-antigen interaction specificity, we performed fluorescence microscopy using EGFP-tagged ORF208 (ORF208-EGFP). The fusion protein exhibited strong binding to wild-type SE006 and ATCC 35150 strains, as well as their O-antigen-complemented strains (Fig. 5D). In contrast, fluorescence was not detected in O-antigen-deficient mutants (Δ*rfaL*). These observations indicate that ORF208 specifically interacts with LPS O-antigen.

### Interaction of ORF208 proteins with LPS

**Fig 6:**
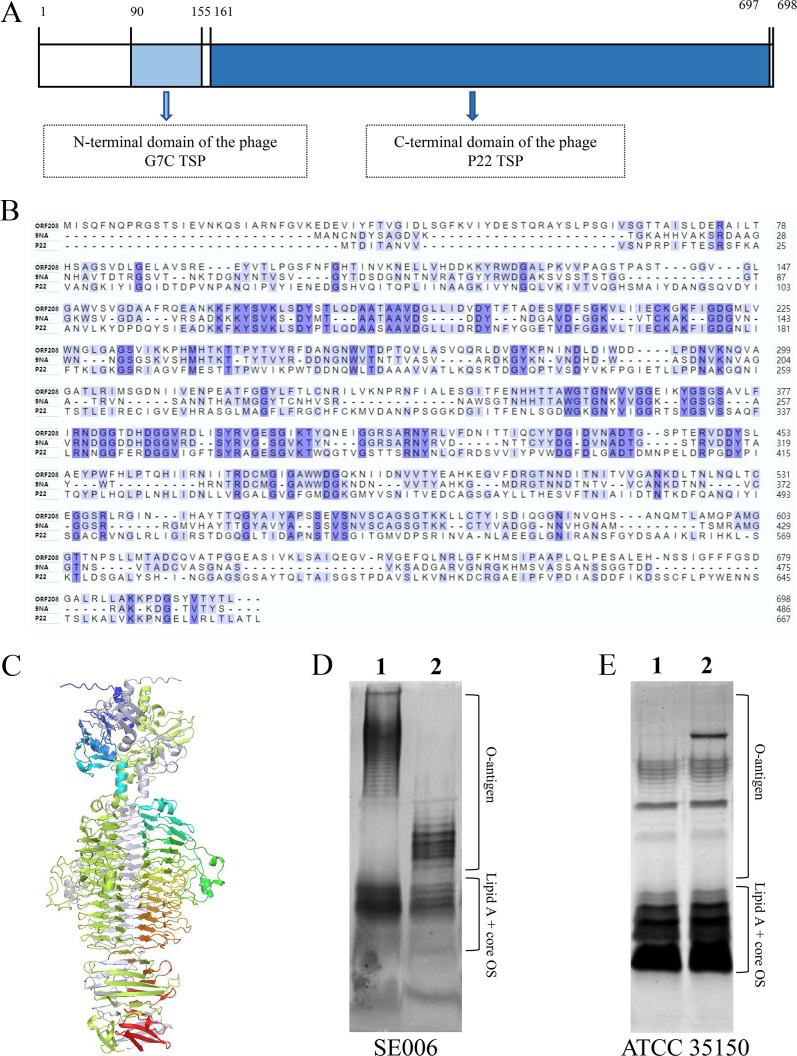
Analysis and identification of the interaction mechanism of ORF208 protein with LPS receptor**.** (**A**) Domain architecture of ORF208. N-terminal domain of the phage G7C tail spike protein (TSP) is located at 90–155 aa and C-terminal domain of the phage P22 TSP is located at 161–697 aa. (**B**) Sequence alignment of ORF208 with tail spike proteins of phage P22 and 9NA. (**C**) The model of the ORF208 trimer was constructed using AlphaFold 2.0. Three monomers are each labeled with a different color. (**D, E**) LPS digestion assay. Lane 1, LPS from *S*. Enteritidis SE006 (**D**) or *E. coli* ATCC 35150 (**E**) incubated with heat-inactivated ORF208. Lane 2: LPS treated with active ORF208. The positions of the fully synthesized LPS with attached O-antigen and lipid A-core oligosaccharide (OS) precursor are indicated.

Bioinformatic analysis revealed ORF208 as a 2,097 bp gene encoding a 698-amino-acid protein. Conserved domain prediction identified two characteristic motifs: an N-terminal domain of the phage G7C tail spike protein (located at residues 90–155) (30) and a C-terminal domain of the phage P22 tail spike protein (located at residues 161-697) (31) (Fig. 6A). Multiple sequence alignment demonstrated 32% and 51% homology between ORF208 and the tail spike proteins (TSPs) of phage P22 and 9NA, respectively (Fig. 6B). Notably, these reference proteins possess receptor-destroying endoglycosidase (endorhamnosidase) activity, enabling binding and hydrolysis of repetitive O-antigen structures in *Salmonella* LPS (9, 32). Structural modeling of ORF208 using AlphaFold predicted a parallel homotrimeric conformation with a large *β*-helical domain, similar to P22 and 9NA TSPs (Fig. 6C). These structural and functional parallels strongly suggest ORF208 may share a conserved receptor recognition mechanism with P22 and 9NA TSPs.

To verify whether ORF208 protein has the same function, we assessed its ability to degrade LPS extracted from SE006 or ATCC 35150 strains using 15% silver-stained SDS-PAGE. The results demonstrated that ORF208 specifically hydrolyzed the long O-antigen chains in the LPS of the SE006 strain, as evidenced by the disappearance of long-chain bands and the concomitant accumulation of short-chain fragments (Fig. 6D). In contrast, no evident degradation of the long-chain O-antigen was observed in the LPS from the ATCC 35150 strain (Fig. 6E). These findings suggest that the functional properties of the ORF208 protein may differ from those of the TSPs of phages 9NA and P22.

## DISCUSSION

The high host specificity of phages represents a key feature distinguishing them from antibiotics, acting as a double-edged sword. On one hand, this high specificity enables phages to selectively kill target bacteria without disrupting commensal microbiota (33). On the other hand, it restricts their host range, thereby severely limiting their applicability (34). Consequently, phages capable of infecting a wide range of important target bacteria are considered ideal candidates for the development of phage antimicrobial agents (35). In this study, we report the isolation and infection mechanisms of GSP004, a polyvalent broad-host-range phage demonstrating infectivity against multiple *Salmonella* serovars and *E. coli* O157:H7. Morphological characterization via TEM revealed a canonical icosahedral capsid with a contractile tail, morphologically consistent with the *Myoviridae* family. Comparative genomic analysis, including whole-genome alignment and phylogenetic tree construction, classified GSP004 within the *Kuttervirus* genus. Phage GSP004 exhibited 98.37% average nucleotide identity with polyvalent *Salmonella* phage BSP101, 98.90% with phage LPEK22, 97.60% with phages GG32, and 97.28% with SenALZ1 (36–39) (Fig. 1D). Despite this genomic conservation, the molecular mechanisms that determine its cross-generic host range (*Salmonella* spp. and *E. coli* O157:H7) remain unknown. Elucidating the mechanisms underlying the intergeneric infectivity of such polyvalent phages toward specific hosts is critical, as this knowledge will enable rational engineering of phage-based antimicrobials and targeted therapeutic biocide development (40).

Phage infection of host bacteria begins with the specific binding of phage-encoded RBPs to host surface receptors (41). The identification of phage-targeted receptors is the first step in the study of phage-host interactions (42). Potential receptors recognized by the phage include structures such as polysaccharides or OMPs exposed on the bacterial surface (43). Here, we demonstrate that the LPS O-antigen is a receptor necessary for GSP004 adsorption and successful infection of *S.* Enteritidis SE006 and *E. coli* ATCC 35150, which does not require any OMP receptor (Fig. 3). It has the same receptor as a similar phage, BSP101, which was previously reported but not studied in greater depth (39). Conventionally, phage adsorption processes require a combination of multiple receptors, including reversible and irreversible adsorption receptors (specific binding) (43, 44). When a phage binds to a specific receptor, the phage then injects nucleic acid into the host cell, which is the beginning of phage-initiated infection. Employing a methodology established by Andres et al. (8) to trigger phage nucleic acid release *in vitro* using a specific receptor, we confirmed that the O-antigen alone could induce GSP004 nucleic acid ejection. This finding indicates that the O-antigen is the sole receptor for phage GSP004 infecting SE006 and ATCC 35150 host bacteria. As with reported phages P22 (8), CBA120 (45), and Det7 (27), they are typically O-antigen-specific phages that are unable to infect O-antigen-deficient bacterial strains as hosts and are also unable to release nucleic acid upon interaction with O-antigen-truncated LPSs.

Typically, O-antigen structures are highly diverse, while the core oligosaccharide structure is relatively conserved at the species level (28). As a result, phages utilizing O-antigens as receptor targets generally display a narrower host range, whereas those employing core oligosaccharide as receptors exhibit broader host range (43). However, certain O-antigen-targeting phages are able to recognize different receptors via multiple RBPs to broaden the host range. For instance, *E. coli* phage CBA120 encodes four tail spike proteins (TSPs) that recognize distinct O-antigens of *E. coli* and *Salmonella*, respectively. Similarly, phages SP6 and K1-5 possess two rotatable TSPs to facilitate alternative host recognition (46, 47). Intriguingly, phage GSP004 maintains a broad host range despite relying on LPS O-antigen as its primary receptor. To elucidate the way phage GSP004 recognizes multiple hosts, phage RBP was identified.

The tail proteins of phages infecting Gram-negative bacteria frequently function as RBPs that mediate adsorption to LPS (48). Bioinformatics analysis revealed that phage GSP004 encodes four tail-associated proteins (ORF206, ORF207, ORF208, and ORF209). We initially hypothesized that at least two of these RBPs were involved in phage recognition of *Salmonella* and *E. coli* O-antigen receptors. However, our results show that the tail protein ORF208 alone binds specifically to the LPS O-antigen of SE006 and ATCC 35150 strains, suggesting that phage GSP004 utilizes a single RBP for receptor recognition.

Further analyses revealed that the ORF208 protein may have the same receptor recognition mechanism as the TSPs of phages P22 and 9NA (9, 32). These phages recognize and hydrolyze the long O-antigen chains of LPS through their TSPs without requiring secondary receptors. However, the results of this study demonstrate that ORF208 exhibits hydrolytic activity only toward the long O-antigen chain in the LPS of SE006 strain, but not toward those in the LPS of ATCC 35150 strain (Fig. 6D and E). This discrepancy suggests that phage GSP004 may employ two distinct molecular mechanisms during the infection of *Salmonella* and *E. coli*. During the lysis of *Salmonella*, ORF208 may function similarly to the TSPs of P22/9NA phages by recognizing and hydrolyzing the long O-antigen chains of LPS to initiate infection. In contrast, during the infection of *E. coli*, ORF208 may initiate infection via an alternative mechanism. It has been reported that phage G7C specifically deacetylates the O-antigen of *E. coli* 4s while leaving the rest of its structure unchanged (30). Based on this, we hypothesize that in the process of infecting *E. coli*, ORF208 may initiate infection via a mechanism analogous to that of phage G7C, involving specific chemical modifications to the O-antigen rather than direct degradation.

In conclusion, this study demonstrates that the polyvalent phage GSP004 utilizes its tail protein ORF208 to recognize and bind to the LPS O-antigen of *Salmonella* and *E. coli*, respectively, but employs distinct molecular strategies to achieve cross-genus infection. These findings offer new insights into the diversity of molecular mechanisms underlying cross-genus infection by polyvalent phages.

## Data Availability

The complete genome sequence of phage GSP004 has been deposited in GenBank and assigned accession number: PP533474.1.

## SUPPLEMENTAL MATERIAL

Supplemental material jvi.00810-25-s0001.docx Figures S1 and S2; Tables S1 to S7.

Table S8 jvi.00810-25-s0002.xlsx GSP004 genome annotation.

ASM does not own the copyrights to Supplemental Material that may be linked to, or accessed through, an article. The authors have granted ASM a non-exclusive, world-wide license to publish the Supplemental Material files. Please contact the corresponding author directly for reuse.
