# Supplementary material for: Polyvalent phage GSP004 recognizes O-antigen polysaccharide receptors in Salmonella and Escherichia coli through tail fiber protein ORF208
Source: J Virol. 2025 Nov 18;99(12):e00810-25. doi: 10.1128/jvi.00810-25 (PMC12724128; doi:10.1128/jvi.00810-25)
Supplement: Supplemental material — Figures S1 and S2; Tables S1 to S7. [file jvi.00810-25-s0001.docx]

**Polyvalent phage GSP004 recognizes O-antigen polysaccharide receptors in *Salmonella* and *Escherichia coli* through tail fiber protein ORF208**

Dongyang Gao^1, 2, 3, ＃^*,* Shenyu Pang^1, ＃^, Yuanhang Zhao^1^, Shunyuan Pan^1^, Xiangyu Kong^1^, Jun Song^1, 2, 3, *^, Dongbo Sun^1, 2, 3, *^

**Supplementary Information**

**Supplementary Figures 1-2.**

**Supplementary Tables 1-7.**

**Supplementary Figures and legends**

**
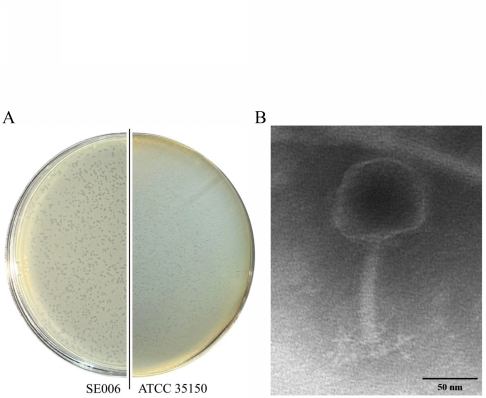
**

**Supplementary Fig. 1. Morphology of phage GSP004.** (A) The plaques formed by phage GSP004 on the lawns of *S*. Enteritidis SE006. (B) TEM of phage GSP004. Scale bar, 50 nm.


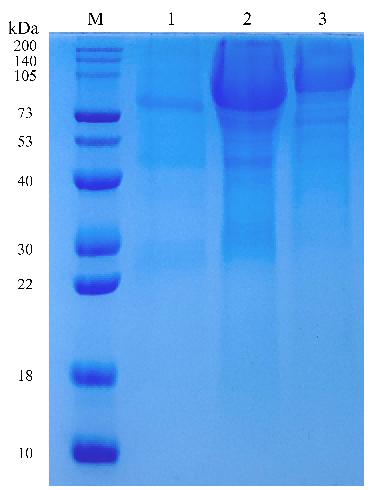


**Supplementary Fig. 2. Purification of recombinant ORF206, ORF207, and ORF209 protein analyzed by SDS-PAGE with Coomassie blue staining.** Lane M: Precision Plus Protein Dual Color Protein Marker (Bio-Rad). Lane 1, purified recombinant ORF206 protein. Lane 2, purified recombinant ORF207 protein. Lane 3, purified recombinant ORF209 protein.

**Supplementary Tables**

**Table S1. Host range was determined by the EOP.**

| **No.** | **Bacterial strain** | **Strain no.** | **Host range** |  | **No.** | **Bacterial strain** | **Strain no.** | **Host range** |
| --- | --- | --- | --- | --- | --- | --- | --- | --- |
| 1 | *S.* Enteritidis | SE006 | **+++** |  | 50 | *S*. Anatis | Sa1001 | **-** |
| 2 |  | SaE001 | **+++** |  | 51 | *S*. Motudi | Sa1002 | **-** |
| 3 |  | SaE002 | **+++** |  | 52 | *S*. Eastbourne | Sa1003 | **-** |
| 4 |  | SaE003 | **+++** |  | 53 | *S*. Stanley | Sa1004 | **+** |
| 5 |  | SaE004 | **++** |  | 54 | *S*. Chester | Sa1005 | **+** |
| 6 |  | SaE005 | **++** |  | 55 | *S*. Newland | Sa1006 | **-** |
| 7 |  | SaE006 | **+++** |  | 56 | *S.* Infantis | Sa1007 | **+** |
| 8 |  | SaE007 | **+++** |  | 57 | *S.* Dublin | CICC 21497 | **-** |
| 9 |  | SaE008 | **+++** |  | 58 | *S.* Cholerasuis | ATCC 10708 | **-** |
| 10 |  | SaE009 | **+++** |  | 59 | *S*. Para-typhi C | CMCC 50118 | **+** |
| 11 |  | SaE010 | **+++** |  | 60 | 1. *coli* | ATCC 35150 O157:H7 | **++** |
| 12 |  | ATCC 13076 | **+++** |  | 61 |  | EDL 933 O157:H7 | **++** |
| 13 | *S*. Typhimurium | SaT001 | **+++** |  | 62 |  | ATCC 43895 O157:H7 | **+** |
| 14 |  | SaT002 | **++** |  | 63 |  | ATCC 43889 O157:H7 | **+** |
| 15 |  | SaT003 | **++** |  | 64 |  | EC001 O157:H7 | **+** |
| 16 |  | SaT004 | **++** |  | 65 |  | EH001 O157:H7 | **+** |
| 17 |  | SaT005 | **+++** |  | 66 |  | EH002 O157:H7 | **++** |
| 18 |  | SaT006 | **+++** |  | 67 |  | EH003 O157:H7 | **+** |
| 19 |  | SaT007 | **-** |  | 68 |  | EH004 O157:H7 | **++** |
| 20 |  | SaT008 | **+** |  | 69 |  | EH005 O157:H7 | **+** |
| 21 |  | SaT009 | **+++** |  | 70 |  | EH006 O157:H7 | **++** |
| 22 |  | SaT010 | **-** |  | 71 |  | EH007 O157:H7 | **+** |
| 23 |  | SaT011 | **-** |  | 72 |  | EH008 O157:H7 | **++** |
| 24 |  | SaT012 | **+++** |  | 73 |  | EH009 O157:H7 | **+** |
| 25 |  | SaT013 | **++** |  | 74 |  | EH010 O157:H7 | **+** |
| 26 |  | SaT014 | **++** |  | 75 |  | EH011 O157:H7 | **+** |
| 27 |  | SaT015 | **+++** |  | 76 |  | EH012 O157:H7 | **+** |
| 28 |  | SaT016 | **++** |  | 77 |  | EH013 O157:H7 | **++** |
| 29 |  | SaT017 | **++** |  | 78 |  | EH014 O157:H7 | **+** |
| 30 |  | SaT018 | **+++** |  | 79 |  | EH015 O157:H7 | **++** |
| 31 |  | SaT019 | **-** |  | 80 |  | EH016 O157:H7 | **+** |
| 32 |  | SaT020 | **+++** |  | 81 |  | MG 1655 | **-** |
| 33 |  | SaT021 | **++** |  | 82 |  | CICC 10667 | **-** |
| 34 |  | SaT022 | **++** |  | 83 |  | ATCC 25922 | **-** |
| 35 |  | SaT023 | **+++** |  | 84 |  | BL21 | **-** |
| 36 |  | ATCC 14028 | **+++** |  | 85 |  | DH5α | **-** |
| 37 |  | CMCC 50115 | **+++** |  | 86 | *Staphylococcus aureus* | ATCC 25923 | **-** |
| 38 |  | CVCC 541 | **+++** |  | 87 | *Klebsiella pneumoniae* | ATCC 700603 | **-** |
| 39 |  | CVCC 542 | **++** |  | 88 | *Proteus mirabilis* | CMCC 49005 | **-** |
| 40 |  | SL 1344 | **-** |  | 89 | *Pseudomonas aeruginosa* | ATCC 27853 | **-** |
| 41 |  | CMCC 50071 | **-** |  | 90 | *Listeria monocytogenes* | ATCC 13932 | **-** |
| 42 | *S*. Pullorum | SaP001 | **+++** |  | 91 | *Listeria monocytogenes* | ATCC 19117 | - |
| 43 |  | SaP002 | **+++** |  |  |  |  |  |
| 44 |  | SaP003 | **+++** |  |  |  |  |  |
| 45 |  | SaP004 | **+++** |  |  |  |  |  |
| 46 |  | SaP005 | **+++** |  |  |  |  |  |
| 47 |  | SaP006 | **+++** |  |  |  |  |  |
| 48 |  | SaP007 | **+++** |  |  |  |  |  |
| 49 |  | SaP008 | **+++** |  |  |  |  |  |

Host range was determined by the efficiency of plating (EOP). +++, 0.5 ≤ EOP ≤ 1.5; ++, 0.01 < EOP < 0.5, +, 0.0001 ≤ EOP ≤ 0.01; -, Not susceptible to phages.

**Table S2 Predicted phage GSP004 encodes tail-associated protein that may act as an RBP.**

| **ORF** | **Putative conserved domains** | **Predicted function** | **Length（aa）** |
| --- | --- | --- | --- |
| ORF206 | Tail_spike_N (pfam18668) | Tail spike protein | 681 |
| ORF207 | Phage_tailspike_middle (cd20481) | Tail spike protein | 927 |
| ORF208 | Phage P22-tail (pfam09251) | Tail spike protein | 699 |
| ORF209 | Tail_spike_N (pfam18668) | Tail spike protein | 993 |

**Table S3. Primers used in this study**

| **ORF** | **Primer designation** | **Primer sequence (5'→3')** | **Product size (bp)** |
| --- | --- | --- | --- |
| ORF206 | pET-28a-ORF206-F | CCGGAATTCATGAACGAAATGTTTAGTCAAGGTG | 2,040 |
|  | pET-28a-ORF206-R | CCGCTCGAGTTTTAGAATTACATTAAATACCGCATC |  |
| ORF207 | pET-28a-ORF207-F | CCGGAATTCATGGGGTATTTTCAAATGACCAGAA | 2,778 |
|  | pET-28a-ORF207-R | CCGCTCGAGAATAGAAGAATCCAATATACGGTAAC |  |
| ORF208 | pET-28a-ORF208-F | CCGGAATTCATGATTTCTCAATTCAATCAACCACGC | 2,094 |
|  | pET-28a-ORF208-R | CCGCTCGAGAAGTGTGTATGTTACATAACTTCC |  |
| ORF209 | pET-28a-ORF209-F | CCGGAATTCATGGCCAACAAACCAACACAG | 2,976 |
|  | pET-28a-ORF209-R | CCGCTCGAGCACGTAAGCACTCTCTAACTTAC |  |
| ORF208-EGFP | pET-28a-EGFP-F | CGCGGATCCATGGTGAGCAAGGGCGAGGAGCT | 717 |
|  | pET-28a-EGFP-R | CCGGAATTCCTTGTACAGCTCGTCCATGCCGAGAG |  |

Note: The solid lines indicate the restriction enzyme cutting sites respectively.

**Table S4. Knockout strain used in this study.**

| **Gene knockout strain** | **Complementary strains** | **Plasmids used** | **Source of strains** |
| --- | --- | --- | --- |
| SE006, Δ*rfaL*::kan^r^ | SE006, Δ*rfaL*::kan^r^ (pHSG396-*rfaL*) | pKD46, Cm^r^, pKD4, pHSG396 | (Gao et al., 2022)，Lab stock |
| SE006, Δ*rfaC*::kan^r^ | SE006, Δ*rfaL*::kan^r^ (pHSG396-*rfaC*) |  |  |
| ATCC 35150, Δ*rfaL*::kan^r^ | ATCC 35150, Δ*rfaL*::kan^r^ (pHSG396-*rfaL*) |  | Lab stock |
| ATCC 35150, Δ*rfaC*::kan^r^ | ATCC 35150, Δ*rfaC*::kan^r^ (pHSG396-*rfaC*) |  |  |

These gene knockout strains were constructed using the lambda Red recombinase system in the previous study.

**Table S5 The *Salmonella* strains used in this study.**

| **Strain** | **Strain ID number** | **Source of strains** |
| --- | --- | --- |
| *Salmonella enterica* serovar [Enteritidis](https://www.ncbi.nlm.nih.gov/nuccore/CP082726.1) (*S.* Enteritidis) | SE006 | (Gao et al 2022)，Lab Stock |
|  | SaE001 | chicken, Lab Stock |
|  | SaE002 | chicken, Lab Stock |
|  | SaE003 | chicken, Lab Stock |
|  | SaE004 | chicken, Lab Stock |
|  | SaE005 | chicken, Lab Stock |
|  | SaE006 | chicken, Lab Stock |
|  | SaE007 | chicken, Lab Stock |
|  | SaE008 | chicken, Lab Stock |
|  | SaE009 | chicken, Lab Stock |
|  | SaE010 | chicken, Lab Stock |
|  | ATCC 13076 | ATCC, Lab Stock |
| *Salmonella enterica* serovar Typhimurium (*S.* Typhimurium) | SaT001 | chicken, Lab Stock |
|  | SaT002 | chicken, Lab Stock |
|  | SaT003 | chicken, Lab Stock |
|  | SaT004 | chicken, Lab Stock |
|  | SaT005 | chicken, Lab Stock |
|  | SaT006 | chicken, Lab Stock |
|  | SaT007 | chicken, Lab Stock |
|  | SaT008 | chicken, Lab Stock |
|  | SaT009 | pig, Lab Stock |
|  | SaT010 | pig, Lab Stock |
|  | SaT011 | pig, Lab Stock |
|  | SaT012 | pig, Lab Stock |
|  | SaT013 | pig, Lab Stock |
|  | SaT014 | pig, Lab Stock |
|  | SaT015 | pig, Lab Stock |
|  | SaT016 | pig, Lab Stock |
|  | SaT017 | pig, Lab Stock |
|  | SaT018 | pig, Lab Stock |
|  | SaT019 | pig, Lab Stock |
|  | SaT020 | pig, Lab Stock |
|  | SaT021 | pig, Lab Stock |
|  | SaT022 | pig, Lab Stock |
|  | SaT023 | pig, Lab Stock |
|  | ATCC 14028 | ATCC, Lab Stock |
|  | CMCC 50115 | CMCC,Lab Stock |
|  | CVCC 541 | CVCC, Lab Stock |
|  | CVCC 542 | CVCC, Lab Stock |
|  | SL 1344 | Lab Stock |
|  | CMCC 50071 | CMCC,Lab Stock |
| *Salmonella enterica* serovar Pullorum (*S.* Pullorum) | SaP001 | chicken, Lab Stock |
|  | SaP002 | chicken, Lab Stock |
|  | SaP003 | chicken, Lab Stock |
|  | SaP004 | chicken, Lab Stock |
|  | SaP005 | chicken, Lab Stock |
|  | SaP006 | chicken, Lab Stock |
|  | SaP007 | chicken, Lab Stock |
|  | SaP008 | chicken, Lab Stock |
| *Salmonella enterica* serovar Anatis (*S.* Anatis) | Sal001 | pork, Lab Stock |
| *Salmonella enterica* serovar Motudi (*S.* Motudi) | Sal002 | pork, Lab Stock |
| *Salmonella enterica* serovar Eastbourne (*S.* Eastbourne) | Sal003 | pork, Lab Stock |
| *Salmonella enterica* serovar Stanley (*S.* Stanley) | Sal004 | pork, Lab Stock |
| *Salmonella enterica* serovar Chester (*S.* Chester) | Sal005 | pork, Lab Stock |
| *Salmonella enterica* serovar Newland (*S.* Newland) | Sal006 | pork, Lab Stock |
| *Salmonella enterica* serovar Infantis (*S.* Infantis) | Sal007 | chicken meat, Lab Stock |
| *Salmonella enterica* serovar Dublin (*S.* Dublin) | CICC 21497 | CICC, Lab Stock |
| *Salmonella enterica* serovar Cholerasuist (*S.* Cholerasuist) | ATCC 10708 | ATCC, Lab Stock |
| *Salmonella enterica* serovar Para-typhi C (*S.* Para-typhi C) | CMCC 50118 | CMCC , Lab Stock |

ATCC: American Type Culture Collection; CMCC: National Center for Medical Culture Collections; CICC: China Center of Industrial Culture Collection; CVCC: National Center for Veterinary Culture Collection; NCTC: National Counterterrorism Center.

**Table S6 The *E. coli* and other bacterial strains used in this study.**

| **Strain** | **Strain ID number** | **Source of strains** |
| --- | --- | --- |
| *Escherichia coli* (*E. coli*) | ATCC 35150 O157:H7 | ATCC, Lab Stock |
|  | EDL 933 O157:H7 | Lab Stock |
|  | ATCC 43895 | ATCC, Lab Stock |
|  | ATCC 43889 | ATCC, Lab Stock |
|  | EC001 O157:H7 | Lab Stock |
|  | EH001 O157:H7 | Lab Stock |
|  | EH002 O157:H7 | Lab Stock |
|  | EH003 O157:H7 | Lab Stock |
|  | EH004 O157:H7 | Lab Stock |
|  | EH005 O157:H7 | Lab Stock |
|  | EH006 O157:H7 | Lab Stock |
|  | EH007 O157:H7 | Lab Stock |
|  | EH008 O157:H7 | Lab Stock |
|  | EH009 O157:H7 | Lab Stock |
|  | EH010 O157:H7 | Lab Stock |
|  | EH011 O157:H7 | Lab Stock |
|  | EH012 O157:H7 | Lab Stock |
|  | EH013 O157:H7 | Lab Stock |
|  | EH014 O157:H7 | Lab Stock |
|  | EH015 O157:H7 | Lab Stock |
|  | EH016 O157:H7 | Lab Stock |
|  | MG 1655 | Lab Stock |
|  | CICC 10667 | CICC, Lab Stock |
|  | ATCC 25922 | ATCC, Lab Stock |
|  | BL21 | Lab Stock |
|  | DH5α | Lab Stock |
| *Staphylococcus aureus* | ATCC 25923 | ATCC, Lab Stock |
| *Klebsiella pneumoniae* | ATCC 700603 | ATCC, Lab Stock |
| *Proteus mirabilis* | CMCC 49005 | CMCC, Lab Stock |
| *Pseudomonas aeruginosa* | ATCC 27853 | ATCC, Lab Stock |
| *Listeria monocytogenes* | ATCC 13932 | ATCC, Lab Stock |
| *Listeria monocytogenes* | ATCC 19117 | ATCC, Lab Stock |

ATCC: American Type Culture Collection; CMCC: National Center for Medical Culture Collections; CICC: China Center of Industrial Culture Collection; CVCC: National Center for Veterinary Culture Collection; NCTC: National Counterterrorism Center.

**Table S7 Primers used for the identification of *Salmonella* and *E. coli* O157.**

| **Genes** | **Primer sequence (5'→3')** | **Species/**  **Serotype** | **Product size (bp)** | **Reference** |
| --- | --- | --- | --- | --- |
| *invA* | F: AAACCTAAAACCAGCAAAGG | *Salmonella enterica* | 605 | (Akiba et al 2011) |
|  | R: TGTACCGTGGCATGTCTGAG |  |  |  |
| *E. coli*  16S rRNA | F: GAAGCTTGCTTCTTTGCT | *E. coli* | 544 | (Sabat et al 2000) |
|  | R: GAGCCCGGGGATTTCACAT |  |  |  |
| *rfbE* | F: CAGGTGAAGGTGGAATGGTTGTC | *E. coli* O157 antigen | 296 | (Bertrand et al 2007) |
|  | R: TTAGAATTGAGACCATCCAATAAG |  |  |  |
| *fliC* | F: AGCTGCAACGGTAAGTGATTT | *E. coli* H7 antigen | 949 | (Bai et al 2010) |
|  | R: GGCAGCAAGCGGGTTGGTC |  |  |  |
| *stx1* | TGCGCACTGAGAAGAAGAGA | Shiga toxin 1 | 655 | (Bai et al 2010) |
|  | CCATGACAACGGACAGCAGTT |  |  |  |
| *stx2* | CCATGACAACGGACAGCAGTT | Shiga toxin 2 | 477 | (Bai et al 2010) |
|  | TGTCGCCAGTTATCTGACATTC |  |  |  |

**References**

1. Akiba M, Kusumoto M, Iwata T. Rapid identification of Salmonella enterica serovars, Typhimurium, Choleraesuis, Infantis, Hadar, Enteritidis, Dublin and Gallinarum, by multiplex PCR. J Microbiol Methods, 2011, 85: 9-15.

2. Bai J, Shi X, Nagaraja TG. A multiplex PCR procedure for the detection of six major virulence genes in Escherichia coli O157:H7. J Microbiol Methods, 2010, 82: 85-89.

3. Bertrand R, Roig B. Evaluation of enrichment-free PCR-based detection on the rfbE gene of Escherichia coli O157--application to municipal wastewater. Water Res, 2007, 41: 1280-1286.

4. Gao D, Ji H, Wang L, Li X, Hu D, Zhao J, Wang S, Tao P, Li X, Qian P. Fitness Trade-Offs in Phage Cocktail-Resistant Salmonella enterica Serovar Enteritidis Results in Increased Antibiotic Susceptibility and Reduced Virulence. Microbiol Spectr, 2022, 10: e0291422.

5. Sabat G, Rose P, Hickey WJ, Harkin JM. Selective and sensitive method for PCR amplification of Escherichia coli 16S rRNA genes in soil. Appl Environ Microbiol, 2000, 66: 844-849.
